# Evaluation of the quality of cause of death statistics of the last decades in the European Union: Lluís Cirera

**DOI:** 10.1093/eurpub/ckac129.761

**Published:** 2022-10-25

**Authors:** L Cirera, M Ballesta, V Uroz, D Sameron

**Affiliations:** 1 Department of Epidemiology, Consejeria de Sanidad, Murcia, Spain; 2 CIBERESP, Epidemiology and Public Health, Madrid, Spain; 3 Department of Social and Health Sciences, University of Murcia, Murcia, Spain; 4 Center of Legal Medicinen, Ministry of Justice, Almeria, Spain

## Abstract

**Introduction:**

The European Union (EU) do not publish the quality of cause-of-death statistics, when is possible to assess quality by reliability. Quality does not present consolidated indicators. Ill-defined and other inaccurate causes have presented different groupings.

**Objective:**

To assess the quality of official cause-of-death statistics of the European Union by member states from 2006 to 2020.

**Methods:**

Cases and population were from the WHO repertory. We selected causes in EU27 countries by up to fourth code-character of ICD10 -on the EuroStat website were not available-. Case counts were grouped into ill- defined, unspecific, less specific (the latter two, in inaccurate), and judicial (inaccurate external causes) categories, based on literature and expertise. We calculated age-adjusted rates to the Standard European Population by country, sex, period (2006-, 2011- and 2016-2020), and quality category. We tested the Comparative Mortality Ratio (CMR) of each country to the European Union median by a Bayesian approach, at 5% statistical significance. We plotted the rates proportion of each quality category in its all causes.

**Results:**

We included 25 countries. Some did not report all years. Six countries showed >19% for ill-defined causes and 3 member states had <5% in both sex and last period. In inaccurate, for the same time period and sex, average pointed 10% with a range of 3-19%. In the same period, CMR exceeded significantly the EU median in 19 and 18 countries for women and men, respectively; and exceeded in unspecific causes in 12 countries for women and men.

**Discussion:**

Literature showed that incorrect causes of death were random distributed. Probably major causes were biased and underestimated.

**Conclusions:**

Quality of cause of death is a useful indicator of mortality statistics reliability. Quality indicators targeted national gaps across EU. We need a new EU task force on statistics of causes of death in accordance with the XXI century.

**Key messages:**

• Quality indicators of causes of death statistics targeted national gaps across the European Union.

• The statistics of death causes underestimated the main causes of death in the European Union.

